# Assessing the pathogenicity of gut bacteria associated with tobacco caterpillar *Spodoptera litura* (Fab.)

**DOI:** 10.1038/s41598-022-12319-w

**Published:** 2022-05-18

**Authors:** Sarita Devi, Harvinder Singh Saini, Sanehdeep Kaur

**Affiliations:** 1 Department of Zoology, Guru Nanak Dev University, Amritsar, Punjab 143005 India; 2 Department of Microbiology, Guru Nanak Dev University, Amritsar, Punjab 143005 India

**Keywords:** Microbiology, Zoology, Pathogenesis

## Abstract

The symbiotic relationship between insects and gut microbes contributes to their fitness by serving immense range of functions viz*.* nutrition and digestion, detoxification, communication and reproduction etc*.* However, this relationship between insect and gut microbes varies from mutualistic to pathogenic. Gut microbes become pathogenic when the healthy normal microbial composition is perturbed leading to the death of insect host. *Spodoptera litura* (Fab.) is a polyphagous pest that causes significant damage to many agricultural crops. The management of this pest primarily depends upon chemical insecticides which have resulted in development of resistance. Thus in search for alternative strategies, culturable gut bacteria isolated from *S. litura* were screened for insecticidal potential. Among these *Serratia marcescens* and *Enterococcus mundtii* induced higher larval mortality in *S. litura.* The mortality rate increased from 32 to 58% due to *S. marcescens* at concentrations ranging from 2.6 × 10^8^ to 5.2 × 10^9^ cfu/ml and 26 to 52% in case of *E. mundtii* due to increase in concentration from 4.6 × 10^8^ to 6.1 × 10^9^ cfu/ml. Both the bacteria negatively affected the development, nutritional physiology and reproductive potential of insect. The results indicated a change in gut microbial composition as well as damage to the gut epithelial membrane. Invasion of gut bacteria into the haemocoel led to septicaemia and ultimately death of host insect. In conclusion both these gut bacteria may serve as potential biocontrol agents against *S. litura.*

## Introduction

Insects live in a symbiotic relationship with various microbes that play a crucial role in their diversification and evolutionary success^1^. These gut microbes serve an immense range of functions including provision of nutrients, digestion, protection from pathogens, detoxification of secondary plant metabolites, communication and reproduction^1^. Contribution of symbiotic microorganisms in decomposition of cellulose components of plant material has been well documented in termites and grasshoppers^2,3^_._ *Buchnera aphidicola* associated with aphids is known to fulfil the requirement of essential amino acids that are lacking in plant sap^4,5^_._ Similarly *Pseudomonas* species, a predominant member of gut microbiota of coffee berry borer, *Hypothenemus hampei* (Ferrari) help in detoxification of caffeine^6^. The gut microbial composition in *Drosophila melanogaster* (Meigen) determines the mating attractiveness, preferentially with individuals harbouring similar microbiota^7,8^. The interactions between hosts and their microbes can range from mutualistic to pathogenic^9^. The gut bacteria may become opportunistic pathogens at a particular time of challenge due to some physiological or environmental changes that triggers their virulence factor or due to perturbation in the gut microbial diversity^10–12^. Mason et al.^9^ reported that translocation of *Enterococcus* from midgut to haemocoel led to its pathogenic state in *Manduca sexta* (Linnaeus). Similarly the mutualistic or pathogenic nature of *Photorhabdus luminescens* depends on whether it lives in gut or hemolymph of host insect^13^. *Enterobacter cloacae*, a member of gut microflora of *Spodoptera litura* (Fab.), when fed orally to its host showed pathogenicity due to change in gut microbial diversity and abundance of *E. cloacae*^14^. Similarly Cakici et al.^15^ reported the insecticidal potential of *Flavobacterium* sp. and *Klebsiella* sp. isolated from *Spodoptera littoralis* (Boisduval) when tested against same insect host. *Serratia marcescens* isolated from larvae of hazelnut weevil *Curculio dieckmanni* (Faust) has also been documented to induce larval mortality in host insect^16^.

Lepidoptera is one of the most diverse and widespread order of class Insecta. The insects belonging to this order play an important role in ecosystem as pollinators and in the food chain. However, the larval stage of most of these insects is phytophagous and cause destruction to many agricultural plants. *S. litura* commonly known as tobacco caterpillar, is a polyphagous lepidopteran pest of many economically important crops such as cotton, soybean, groundnut, tobacco and vegetables^17^. The control of this pest mainly involves the application of chemical insecticides such as organophosphates, carbamates and synthetic pyrethroids^18,19^. However, many of these insecticides have been found to be ineffective due to development of resistance in this pest to different groups of insecticides^19–22^. Besides development of insecticide resistant populations of insects, the hazardous effects of synthetic insecticides on human health, environment and non-target organisms are also a matter of concern^19,23,24^. Therefore, there is need for alternative ecofriendly strategies for pest management.

The use of pathogenic microbes viz*.* fungi, bacteria, viruses and nematodes are gaining popularity as an alternative strategy to chemical insecticides. Due to their species specificity and environmental safety, these have been exploited to develop insecticide formulations. Among these, *Bacillus thuringiensis* (Bt) has been commercially used as bioinsecticide against insect pests belonging to Diptera, Coleoptera and Lepidoptera. However, reports on development of resistance in lepidopteran pests viz*. Plutella xylostella* (Linnaeus)*, Pectinophora gossypiella* (Saunders), *Spodoptera frugiperda* (JE Smith) and *Helicoverpa zea* (Boddie) towards Bt insecticides has become a matter of concern^25–28^. The resistance to Bt insecticides necessitates the need to explore new niches as sources of novel microorganisms having insecticidal activity. In this respect, as a step towards finding potential candidates for biological control, the present study aimed to determine the pathogenicity of culturable gut microbes associated with *S. litura* infesting crops of this region.

## Results

### Screening bioassays

**Figure 1:**
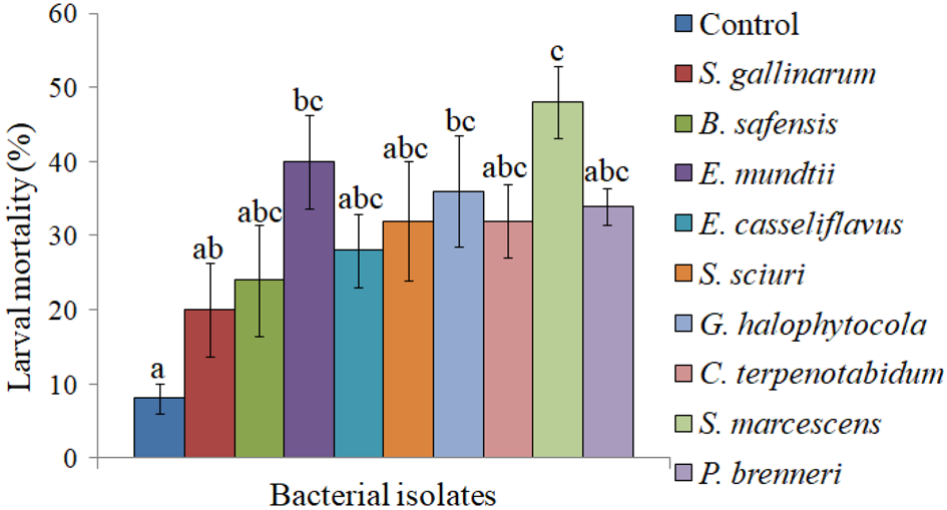
Pathogenicity of bacterial isolates of *S. litura* against its second-instar larvae at 1.8 × 10^9^ cfu/ml (approx). Columns and bars represent the mean ± SE. Different letters above the columns representing each bacteria indicate significant differences at Tukey's test p ≤ 0.05.

Screening of gut bacteria viz. *S. gallinarum*, *B. safensis*, *E. mundtii*, *E. casseliflavus*, *S. sciuri*, *G. halophytocola*, *C. terpenotabidum*, *S. marcescens* and *P. brenneri* for insecticidal activity against *S. litura* indicated significantly higher larval mortality (20–48%) in comparison to control (Fig. 1). Among the tested bacteria, *E. mundtii* and *S. marcescens* exhibited higher larval mortality i.e. 40% and 48%, thus both these bacteria were selected for detailed bioassay studies.

### Dose–response experiments

#### Mortality and development period

**Figure 2:**
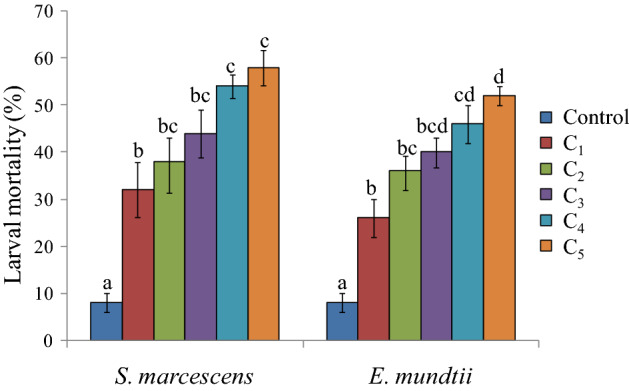
Influence of different concentrations of *S. marcescens* (C_1_ = 2.6 × 10^8^ cfu/ml, C_2_ = 6.4 × 10^8^ cfu/ml, C_3_ = 1.6 × 10^9^ cfu/ml, C_4_ = 3.0 × 10^9^ cfu/ml and C_5_ = 5.2 × 10^9^ cfu/ml) and *E. mundtii* (C_1_ = 4.6 × 10^8^ cfu/ml, C_2_ = 8.9 × 10^8^ cfu/ml, C_3_ = 1.8 × 10^9^ cfu/ml, C_4_ = 3.4 × 10^9^ cfu/ml and C_5_ = 6.1 × 10^9^ cfu/ml) on larval mortality of *S. litura*. Columns and bars represent the mean ± SE. Different letters above the columns represent significant differences at Tukey's test p ≤ 0.05.

**Figure 3:**
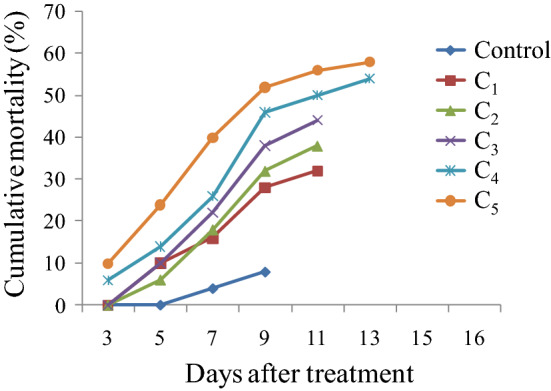
Mean cumulative mortality of second instar larvae of *S. litura* fed on castor leaves treated with different concentrations (C_1_ = 2.6 × 10^8^ cfu/ml, C_2_ = 6.4 × 10^8^ cfu/ml, C_3_ = 1.6 × 10^9^ cfu/ml, C_4_ = 3.0 × 10^9^ cfu/ml and C_5_ = 5.2 × 10^9^ cfu/ml) of *S. marcescens*.

**Figure 4:**
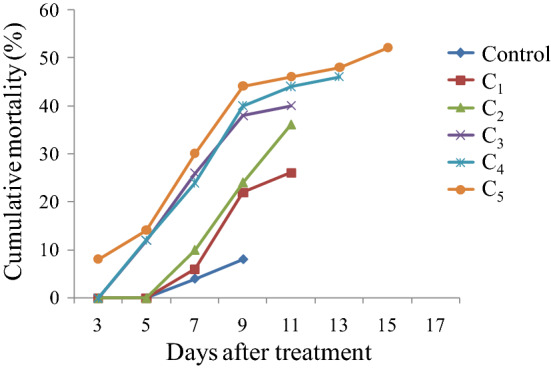
Mean cumulative mortality of second instar larvae of *S. litura* fed on castor leaves treated with different concentrations (C_1_ = 4.6 × 10^8^ cfu/ml, C_2_ = 8.9 × 10^8^ cfu/ml, C_3_ = 1.8 × 10^9^ cfu/ml, C_4_ = 3.4 × 10^9^ cfu/ml and C_5_ = 6.1 × 10^9^ cfu/ml) of *E. mundtii*.

**Figure 5:**
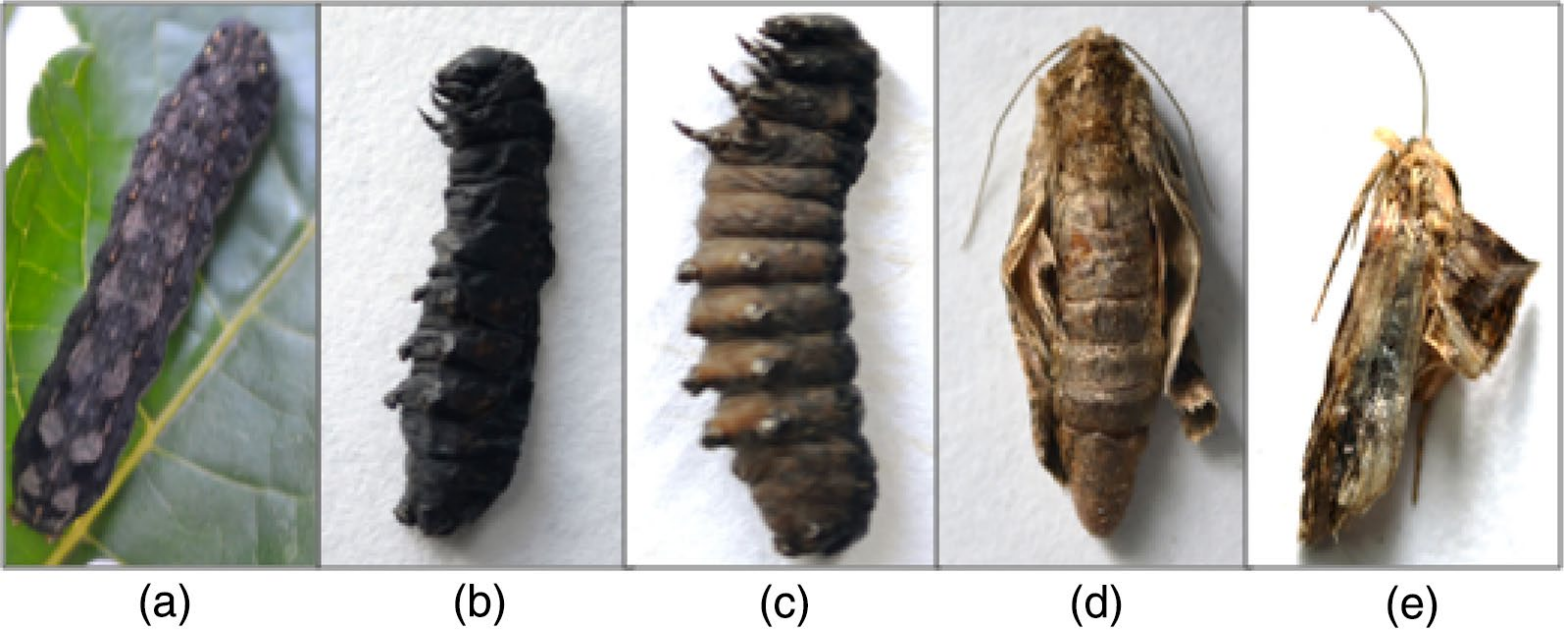
Effect of bacterial infection on *S. litura* (**a**) healthy (control) larva, (**b**, **c**) dead larvae, (**d**, **e**) morphologically deformed adults.

Results presented in Table 1 depict a significant effect of *S. marcescens* and *E. mundtii* on survival and development of *S. litura*. Both the bacteria caused significantly higher larval mortality relative to control. The leaves treated with different concentrations of *S. marcescens* caused 32–58% mortality in *S. litura* larvae (F = 15.20**, p ≤ 0.05) (Fig. 2). The mortality rate increased in a concentration dependent manner. Similar results were obtained due to *E. mundtii* cell suspension that caused 26–52% mortality in *S. litura* larvae (F = 22.64**, p ≤ 0.05) (Fig. 2). The larval mortality started after 3rd day of treatment at higher concentrations (3.0 × 10^9^ cfu/ml and 5.2 × 10^9^ cfu/ml) of *S. marcescens* and continued till 13th day (Fig. 3). Maximum larval deaths were observed with cumulative mortality of 52% at 9th day of treatment. Similarly in case of *E. mundtii,* the larval mortality started after 3rd day of treatment at the highest concentration (6.1 × 10^9^ cfu/ml) and continued for fifteen days (Fig. 4). The LC_50_ values for both the bacteria were calculated using Probit analysis, that came out to be 2.4 × 10^9^ and 5.6 × 10^9^ cfu/ml respectively for *S. marcescens* and *E. mundtii*. Relative to control, the infected larvae showed the symptoms of sluggishness, cessation of feeding and the dead larvae became black in colour, flaccid but with intact integument (Fig. 5a–c).

**Table 1:** Influence of different concentrations of *S. marcescens* and *E. mundtii* on development and adult emergence of *S. litura*.

Bacteria	Concentration (cfu/ml)	Larval period (days)	Pupal period (days)	Total developmental period (days)	Adult emergence (%)	Adult deformities (%)
*S. marcescens*	Control	12.06 ± 0.48^a^	8.90 ± 0.19^a^	20.96 ± 0.40^a^	91.06 ± 4.17^c^	3.20 ± 0.80^a^
	2.6 × 10^8^	12.35 ± 0.26^a^	8.69 ± 0.24^a^	21.04 ± 0.33^a^	86.40 ± 2.24^c^	6.40 ± 1.02^ab^
	6.4 × 10^8^	12.44 ± 0.47^a^	9.11 ± 0.32^ab^	21.55 ± 0.64^ab^	81.60 ± 1.96^bc^	7.60 ± 1.93^ab^
	1.6 × 10^9^	12.88 ± 0.38^a^	9.26 ± 0.12^ab^	22.14 ± 0.28^ab^	82.40 ± 1.20^bc^	11.20 ± 2.51^bc^
	3.0 × 10^9^	13.78 ± 0.54^ab^	9.50 ± 0.20^ab^	23.28 ± 0.70^bc^	72.00 ± 2.70^ab^	16.40 ± 1.96^ cd^
	5.2 × 10^9^	14.88 ± 0.40^b^	10.10 ± 0.33^b^	24.98 ± 0.33^c^	64.80 ± 1.01^a^	20.20 ± 1.77^e^
	F-value	6.05**	3.98**	10.51**	15.47**	13.22**
*E. mundtii*	Control	12.06 ± 0.48^a^	8.90 ± 0.19^a^	20.96 ± 0.40^a^	91.06 ± 4.17^c^	3.20 ± 0.80^a^
	4.6 × 10^8^	12.09 ± 0.14^a^	9.20 ± 0.25^ab^	21.29 ± 0.33^a^	87.40 ± 3.41^bc^	5.00 ± 0.70^ab^
	8.9 × 10^8^	12.40 ± 0.28^a^	9.50 ± 0.44^ab^	21.90 ± 0.42^ab^	84.20 ± 2.17^abc^	5.40 ± 0.67^ab^
	1.8 × 10^9^	14.00 ± 0.63^ab^	9.64 ± 0.26^ab^	23.64 ± 0.51^bc^	78.34 ± 3.57^abc^	8.60 ± 1.28^b^
	3.4 × 10^9^	14.65 ± 0.58^bc^	10.25 ± 0.23^b^	24.90 ± 0.68^ cd^	76.60 ± 2.27^ab^	13.20 ± 1.06^c^
	6.1 × 10^9^	16.09 ± 0.48^c^	10.30 ± 0.30^b^	26.39 ± 0.46^e^	71.20 ± 1.24^a^	13.00 ± 0.70^c^
	F-value	12.39**	3.65**	20.29**	6.15**	22.32**

The values (Mean ± SE) followed by different letters (superscript) with in a column indicate significant differences at Tukey's test p ≤ 0.05, **Significant at 1% level.

Bacterial treatment also influenced the development of insect. The larval period tended to increase but significant effect was observed at higher concentrations (Table 1). At the highest concentration of *S. marcescens*, the larvae took 14.88 days to pupate in comparison to 12.06 days in control (F = 6.05, p ≤ 0.05). The pupal period was also affected at the highest concentration. The overall development period from larva to adult extended significantly at higher concentrations i.e. 3.0 × 10^9^ and 5.2 × 10^9^ cfu/ml where the insect took 23.28 and 24.98 days respectively in comparison to 20.96 days in control (F = 10.51, p ≤ 0.05) (Table 1).

Similar effects were observed due to *E. mundtii* where the larval period prolonged significantly by 2.59 to 4.03 days at higher concentrations i.e. 3.4 × 10^9^ and 6.1 × 10^9^ cfu/ml with respect to control (Table 1). Significant effect was also detected on pupal period that ultimately extended the total development period by 2.68 to 5.43 days at concentrations ranging between 1.8 × 10^9^ to 6.1 × 10^9^ cfu/ml in comparison to control.

#### Adult emergence and reproductive potential

*Serratia marcescens* treatment significantly decreased the adult emergence of *S. litura* at higher concentrations i.e. 3.0 × 10^9^ and 5.2 × 10^9^ cfu/ml (F = 15.47, p ≤ 0.05) (Table 1). Similarly adult emergence tended to decrease when the larvae were fed on cell suspension of *E. mundtii*, however, significant effect was recorded at higher concentrations (3.4 × 10^9^ and 6.1 × 10^9^ cfu/ml) where 76.60 to 71.20% adults emerged as compared to 91.06% in control (F = 6.15, p ≤ 0.05) (Table 1). The bacterial infection also caused morphological deformities in adults such as unequal and crumpled wings (Fig. 5d,e). Except for the lower concentrations, the percentage of morphologically deformed individuals was significantly higher in both the bacterial treatments (*S. marcescens,* F = 13.22, p ≤ 0.05; *E. mundtii, *F = 22.32, p ≤ 0.05) (Table 1).

**Figure 6:**
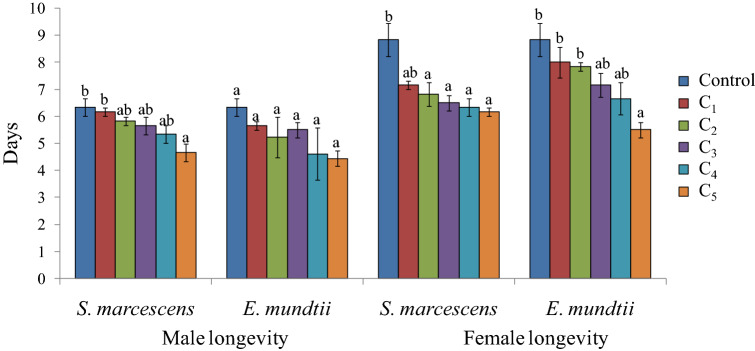
Influence of different concentrations of *S. marcescens* (C_1_ = 2.6 × 10^8^ cfu/ml, C_2_ = 6.4 × 10^8^ cfu/ml, C_3_ = 1.6 × 10^9^ cfu/ml, C_4_ = 3.0 × 10^9^ cfu/ml and C_5_ = 5.2 × 10^9^ cfu/ml) and *E. mundtii* (C_1_ = 4.6 × 10^8^ cfu/ml, C_2_ = 8.9 × 10^8^ cfu/ml, C_3_ = 1.8 × 10^9^ cfu/ml, C_4_ = 3.4 × 10^9^ cfu/ml and C_5_ = 6.1 × 10^9^ cfu/ml) on adult longevity of *S. litura*. Columns and bars represent the mean ± SE. Different letters above the columns represent significant differences at Tukey's test p ≤ 0.05.

**Figure 7:**
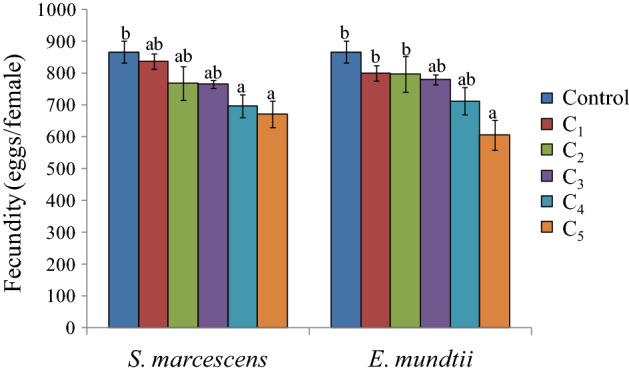
Influence of different concentrations of *S. marcescens* (C_1_ = 2.6 × 10^8^ cfu/ml, C_2_ = 6.4 × 10^8^ cfu/ml, C_3_ = 1.6 × 10^9^ cfu/ml, C_4_ = 3.0 × 10^9^ cfu/ml and C_5_ = 5.2 × 10^9^ cfu/ml) and *E. mundtii* (C_1_ = 4.6 × 10^8^ cfu/ml, C_2_ = 8.9 × 10^8^ cfu/ml, C_3_ = 1.8 × 10^9^ cfu/ml, C_4_ = 3.4 × 10^9^ cfu/ml and C_5_ = 6.1 × 10^9^ cfu/ml) on fecundity of *S. litura*. Columns and bars represent the mean ± SE. Different letters above the columns represent significant differences at Tukey's test p ≤ 0.05.

**Figure 8:**
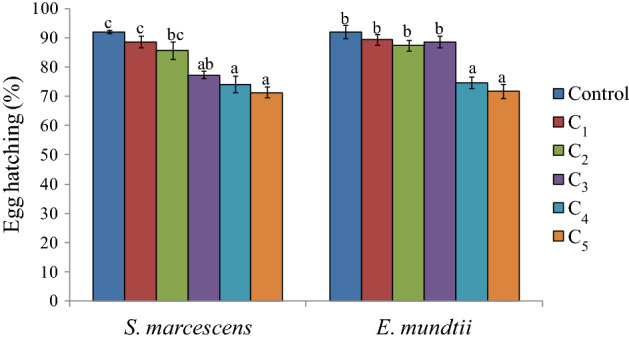
Influence of different concentrations of *S. marcescens* (C_1_ = 2.6 × 10^8^ cfu/ml, C_2_ = 6.4 × 10^8^ cfu/ml, C_3_ = 1.6 × 10^9^ cfu/ml, C_4_ = 3.0 × 10^9^ cfu/ml and C_5_ = 5.2 × 10^9^ cfu/ml) and *E. mundtii* (C_1_ = 4.6 × 10^8^ cfu/ml, C_2_ = 8.9 × 10^8^ cfu/ml, C_3_ = 1.8 × 10^9^ cfu/ml, C_4_ = 3.4 × 10^9^ cfu/ml and C_5_ = 6.1 × 10^9^ cfu/ml) on egg hatching of *S. litura*. Columns and bars represent the mean ± SE. Different letters above the columns represent significant differences at Tukey's test p ≤ 0.05.

The effects of bacterial suspensions were also detected on females which showed reduced longevity. Except for the lowest concentration of *S. marcescens*, the female longevity decreased significantly by 2.0 to 2.67 days in comparison to control (F = 7.15, p ≤ 0.05) (Fig. 6). Significant effect of *S. marcescens* was also observed on male longevity at the highest concentration (F = 4.40, p ≤ 0.05). In case of *E. mundtii* no significant inhibitory effects were detected on adult longevity except for the highest concentration in case of females (F = 5.97, p ≤ 0.05) (Fig. 6). The reproductive potential of females was significantly reduced at higher concentrations (3.0 × 10^9^ and 5.2 × 10^9^ cfu/ml) of *S. marcescens* where the female laid only 696.66 to 671.00 eggs throughout its life as compared to 866.66 eggs in control (F = 4.53, p ≤ 0.05) (Fig. 7). Similarly in case of *E. mundtii,* fecundity was found to be decreased significantly at the highest concentration. The bacterial infection further decreased the viability of eggs with significant effect at higher concentrations (*S. marcescens*, F = 16.35, p ≤ 0.05; *E. mundtii,* F = 17.11, p ≤ 0.05) (Fig. 8).

#### Effect of *S. marcescens* and *E. mundtii* on nutritional physiology

As is evident from Table 2, *S. marcescens* significantly influenced the nutritional indices of *S. litura.* The relative consumption rate of larvae feeding on bacteria treated leaves was significantly decreased which in turn led to decrease in relative growth rate of larvae at all the concentrations (RGR, F = 3.60, p ≤ 0.05; RCR, F = 17.12, p ≤ 0.05). However, no significant difference was found within the different concentrations of bacterial treatments. The ECI value also decreased from 1.82% in control to 1.10–1.00% due to bacterial infection (F = 8.75, p ≤ 0.05). A significant decrease in ECD was observed at 3.0 × 10^9^ and 5.2 × 10^9^ cfu/ml of *S. marcescens* cell suspension (F = 4.82, p ≤ 0.05). Except for the lowest concentration, a significant reduction was also detected in approximate digestibility (F = 5.44, p ≤ 0.05) (Table 2).

**Table 2:** Influence of different concentrations of *S. marcescens* and *E. mundtii* on food consumption and utilization of *S. litura* larvae.

Bacteria	Concentration (cfu/ml)	RGR (mg mg^−1^day^−1^)	RCR (mg mg^−1^day^−1^)	ECI (%)	ECD (%)	AD (%)
*S. marcescens*	Control	0.33 ± 0.040^b^	34.56 ± 0.83^b^	1.82 ± 0.24^b^	8.04 ± 0.86^b^	95.51 ± 1.28^b^
	2.6 × 10^8^	0.25 ± 0.006^a^	24.50 ± 0.77^a^	1.10 ± 0.03^a^	6.45 ± 0.43^ab^	91.35 ± 1.51^ab^
	6.4 × 10^8^	0.26 ± 0.007^a^	25.50 ± 1.19^a^	1.13 ± 0.05^a^	6.13 ± 0.33^ab^	89.53 ± 0.90^a^
	1.6 × 10^9^	0.25 ± 0.008^a^	25.58 ± 0.54^a^	1.08 ± 0.02^a^	6.53 ± 0.21^ab^	89.99 ± 1.22^a^
	3.0 × 10^9^	0.25 ± 0.007^a^	26.68 ± 1.06^a^	1.00 ± 0.05^a^	5.53 ± 0.15^a^	90.20 ± 0.62^a^
	5.2 × 10^9^	0.25 ± 0.006^a^	25.28 ± 0.89^a^	1.00 ± 0.01^a^	5.34 ± 0.19^a^	87.85 ± 0.89^a^
	F-value	F = 3.60**	F = 17.12**	F = 8.75**	F = 4.82**	F = 5.44**
*E. mundtii*	Control	0.33 ± 0.040^b^	34.56 ± 0.83^c^	1.82 ± 0.24^b^	8.04 ± 0.86^b^	95.51 ± 1.28^b^
	4.6 × 10^8^	0.26 ± 0.002^ab^	27.93 ± 1.97^bc^	1.46 ± 0.34^ab^	6.38 ± 0.53^ab^	92.52 ± 0.35^ab^
	8.9 × 10^8^	0.23 ± 0.019^a^	26.33 ± 1.10^abc^	1.45 ± 0.23^ab^	6.04 ± 0.51^ab^	92.29 ± 0.85^ab^
	1.8 × 10^9^	0.22 ± 0.012^a^	14.84 ± 5.02^ab^	1.39 ± 0.24^ab^	5.04 ± 0.63^a^	91.13 ± 0.78^a^
	3.4 × 10^9^	0.22 ± 0.009^a^	14.80 ± 2.19^a^	0.82 ± 0.06^a^	4.93 ± 0.68^a^	91.03 ± 1.21^a^
	6.1 × 10^9^	0.19 ± 0.025^a^	13.83 ± 4.26^a^	0.74 ± 0.07^a^	4.60 ± 0.65^a^	90.73 ± 0.50^a^
	F-value	F = 5.53**	F = 8.48**	F = 3.44**	F = 3.77**	F = 3.88**

The values (Mean ± SE) followed by different letters (superscript) with in a column are significantly different. Tukey's test p ≤ 0.05, **Significant at 1% level.

*RGR* Relative growth rate, *RCR* Relative consumption rate, *ECI* Efficiency of conversion of ingested food, *ECD* Efficiency of conversion of digested food, *AD* Approximate digestibility.

Similar effects of *E. mundtii* were observed on various nutritional parameters of *S. litura* (Table 2). There was a significant drop in relative consumption and growth rate of larvae. With respect to control, the values of RCR decreased by 57.06 to 59.98% with concomitant decrease of 33.33 to 42.42% in RGR at higher concentrations i.e. 1.8 × 10^9^ cfu/ml and 6.1 × 10^9^ cfu/ml (RGR, F = 5.53, p ≤ 0.05; RCR, F = 8.48, p ≤ 0.05). Similarly, the efficiency of conversion of ingested and digested food of larvae decreased significantly by 2.21 to 2.45 and 1.59 to 1.74 times respectively at higher concentrations (ECI, F = 3.44, p ≤ 0.05; ECD, F = 3.77, p ≤ 0.05) (Table 2). Significant negative impact of *E. mundtii* was also detected on approximate digestibility of food at concentrations ranging from 1.8 × 10^9^ cfu/ml to 6.1 × 10^9^ cfu/ml (F = 3.88, p ≤ 0.05) (Table 2).

#### Effect of *S. marcescens* and *E. mundtii* on gut microflora of *S. litura*

As is evident from Table 3 there is considerable difference in gut microbial composition of control and treated larvae. The gut microflora of control larvae consisted of *E. mundtii*, *E. casseliflavus* and *A. hemolyticus* with 7.4 × 10^6^, 6.9 × 10^6^ and 4.0 × 10^5^ cfu/ml respectively. However, the treatment of larvae with *S. marcescens* led to change in bacterial abundance. There was increase in bacterial concentration of *S. marcescens* with 7.9 × 10^7^ cfu/ml relative to other bacterial cultures i.e. *E. mundtii* and *E. casseliflavus* with 4.1 × 10^4^ and 3.6 × 10^4^ cfu/ml respectively (Table 3). Similarly the larvae infected with *E. mundtii* showed the dominance of *E. mundtii* with cfu count of 9.3 × 10^7^ per ml in comparison to 5.6 × 10^4^ cfu/ ml of *E. casseliflavus*. *E. mundtii* was observed in both the treated as well as control larvae, while *A. hemolyticus* was absent in the larvae treated with both the bacterial concentrations.

**Table 3:** Effect of oral ingestion of *S. marcescens* and *E. mundtii* on gut microbial diversity of *S. litura* larvae.

Treatments	Abundance of gut bacteria (cfu/ml)			
	*E. mundtii*	*E. casseliflavus*	*S. marcescens*	*A.hemolyticus*
Control	7.4 × 10^6^	6.9 × 10^6^	–	4.0 × 10^5^
*S. marcescens*	4.1 × 10^4^	3.6 × 10^4^	7.9 × 10^7^	–
*E. mundtii*	9.3 × 10^7^	5.6 × 10^4^	–	–

### Histological analysis

**Figure 9:**
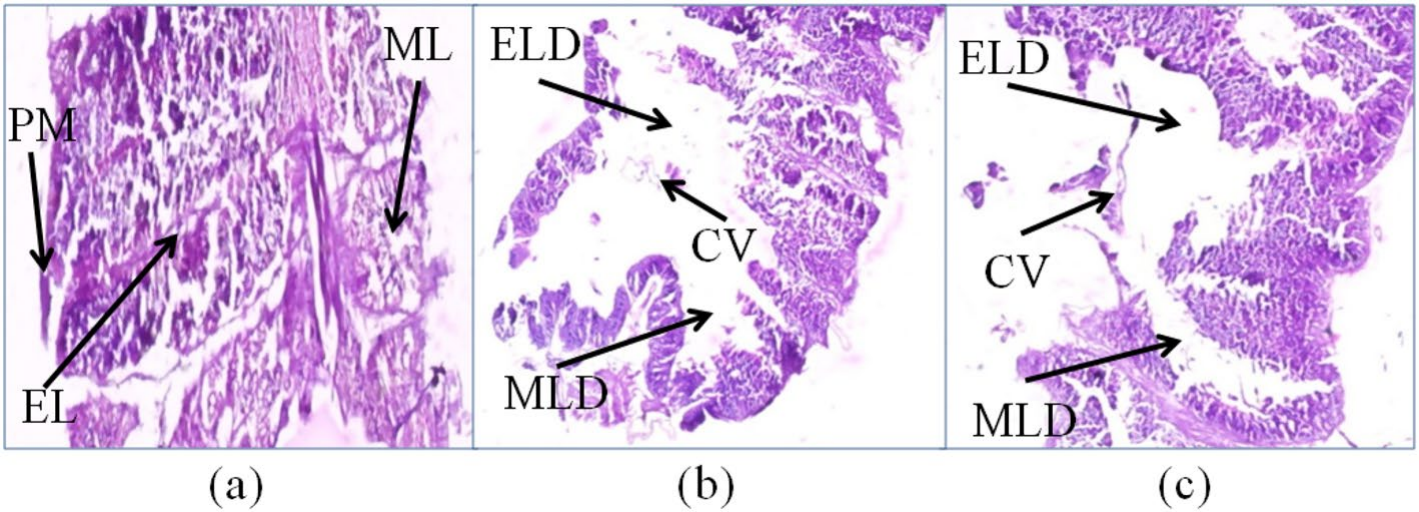
Longitudinal section through the midgut of 4th instar *S. litura* larvae (**a**) control larva fed on untreated diet, (**b**) larva fed on leaves treated with *S. marcescens*, (**c**) larva fed on leaves treated with *E. mundtii*. *PM* Peritrophic membrane, *EL* Epithelial layer, *ML* Muscle layer, *ELD* Epithelial layer disruption, *CV* Cytoplasmic vacuolization, *MLD* Muscle layer disruption.

Difference in the histology of gut of *S. litura* was observed among the control and treated larvae. The midgut cross-sections of larvae fed on cell suspensions of *S. marcescens* and *E. mundtii* showed damage of the midgut epithelial cells with vacuolization of the cytoplasm, brush border membrane and peritrophic membrane destruction (Fig. 9). However, the control larvae showed a well-preserved layer of epithelial cells, peritrophic membrane and muscular layer of the midgut.

### Presence of *S. marcescens* and *E. mundtii* in larval haemolymph

The growth of both the bacteria was observed in the hemolymph of infected larvae due to *S. marcescens* and *E. mundtii* infection, however, no growth was observed in case of control healthy larvae**.**

## Discussion

Gut microbes play an important role in insects ranging from digestion, detoxification, communication and reproduction etc^1^. Besides their functional role the native gut microbes have also been reported to be pathogenic in insects^14,29,30^. In the present study, screening of culturable bacteria associated with larval and pupal stages of *S. litura* indicated higher mortality of host larvae due to *S. marcescens* and *E. mundtii.* The pathogenicity of *S. marcescens* and *E. mundtii* has earlier been reported in various lepidopteran insects viz*. Bombyx mori* (Linnaeus), *Spodoptera exigua* (Hubner)*, Galleria mellonella* (Linnaeus), *Lymantria dispar* (Linnaeus), *Malacosoma neustria* (Linnaeus), *Plodia interpunctella* (Hubner) and *Ephestia kuehniella* (Zeller)^30–36^. Other strains of *Enterococcus *viz*. Enterococcus faecalis* and *Enterococus faecium* have earlier been documented to cause mortality in *S. exigua* and *G. mellonella*^37–39^.

The *S. litura* larvae infected with *S. marcescens* and *E. mundtii* showed the symptoms of lethargy, dark colouration of body, flaccid with intact integument which are typical symptoms of bacterial infection^40^. Likewise *P. interpunctella* and *E. kuehniella* infected with *S. marcescens* showed similar symptoms of infection^32,34,36,41^.

Histopathological studies conducted on *S. litura* infected with *S. marcescens* and *E. mundtii* indicated disruption of peritrophic membrane, damage to epithelial cells and cytoplasmic vacuolization which is similar to earlier report on *S. litura* due to bacterial infection of *S.* *marcescens*^42^. Peritrophic membrane acts as first line of defense in insects against microbial pathogens. Chitin is the main component of peritrophic membrane that lines the midgut epithelium^43^. There are reports documenting the production of toxins and hydrolytic enzymes such as hemolysins, chitinases, proteases, lipases and phospholipases from *S. marcescens* that contribute to its pathogenicity in insects^42,44,45^. The different type of chitinases viz*.* exochitinases, endochitinases and chitobiosidases damage the midgut peritrophic membrane that further help the bacterial invasion into the haemocoel^42,46,47^. *S. marcescens* and *E. mundtii* in our study were observed to grow in the hemolymph of the larvae indicating that the bacteria traversed the intestinal epithelial barrier. The bacterial invasion in hemolymph was also reported in *S. litura, Helicoverpa armigera* (Hubner) and *M. sexta* due to infection of *Serratia* and *Enterococcus* sp.^9,42,48^. The present study is in line with the earlier reports indicating that bacterial proliferation in hemolymph after crossing the intestinal barrier cause septicaemia which ultimately led to the death of its host^49^.

*Serratia* and *Enterococcus* have been known to be the normal flora of larvae, pupae and adults of lepidopteran insects^50–52^. These bacteria are generally found in low numbers in digestive tract and are not pathogenic. However, the bacteria may become pathogenic when the insect immune system gets weakened or due to alterations in gut microbial composition of insects^12,53,54^. Earlier studies revealed that perturbation of gut microbial composition led to the death of host insect^12,14,55–57^. Present study showed the difference in gut microbial composition of control and treated larvae. *Serratia* and *Enterococcus* have been found to increase in numbers in treated *S. litura* larvae with respect to control larvae. It is in line with the previous report on *S. litura* where the *S. marcescens* is able to colonize the midgut tissues after oral infection and there after the population increased as compared to control larvae^42^. *E. mundtii* found in low numbers in gut microflora of healthy larvae, however, increased number of bacterial colonies led to flacherie disease in *B. mori* larvae^32^. *S. marcescens* successfully inhabitated the gut by increasing its number and replacing the other gut associated beneficial microflora in *H. armigera*^48^. The infection due to *S. marcescens* and *E. mundtii* prolonged the development of *S. litura* which to similar to the reports on *S. litura, H. armigera* and *Bactrocera dorsalis* (Hendel) due to infection of *S. marcescens, Enterobacter cloacae and Lactobacillus lactis*^14,46,48,58^. Bacterial infection further affected the nutritional physiology of *S. litura* larvae*.* The significant decrease in growth rate of *S. litura* may be attributed to decreased relative consumption rate. The treated *S. litura* larvae also showed reduction in efficiency of conversion of ingested and digested food as well as approximate digestibility. Previous studies also revealed the inhibitory effects on nutritional physiology of *S. litura* and *Cnaphalocrocis medinalis* (Guenee) due to *E. cloacae* and *B. thuringiensis* infection^14,59^. Chandrasekaran et al.^60^ reported negative effect on nutritional physiology of *S. litura* due to extracellular chitinases produced from *Bacillus subtilis*. Destruction of peritrophic membrane and midgut epithelial cells observed during histopathological studies on *S. litura* may have impaired the digestive functions by interfering with digestive and protective enzymes activity as suggested by Zhang et al.^16^. The decrease in digestive function may further slow the growth of larvae. Reduction in adult emergence, fecundity and egg hatchability was also observed in the bacteria treated groups of *S. litura* larvae. *S. marcescens* was earlier reported to decrease the adult emergence and reproductive potential of *S. litura*^46^. These results indicate that *S. marcescens* and *E. mundtii* act as opportunistic pathogens which also exert growth inhibitory and toxic effects on *S. litura.*

## Conclusion

Present study revealed the insecticidal potential of *S. marcescens* and *E. mundtii*. Both the bacterial isolates showed pathogenicity against second-instar larvae of *S. litura.* The ingestion of bacteria negatively affected the development and nutritional physiology of insect. Both the bacteria after successful establishment started degrading the gut wall and invaded the haemocoel thereby causing the death of the host. In conclusion these results indicate that *S. marcescens* and *E. mundtii* have a potential to be used as biocontrol agent against insect pests.

## Materials and methods

### Mass rearing of insect

The egg masses and larvae of *S. litura* were collected from cabbage and cauliflower fields around Amritsar (Punjab), India. The larvae were reared on fresh castor leaves. The culture was maintained in the laboratory at temperature and humidity conditions of 25 ± 2 °C and 65 ± 5% respectively as per the protocol of Datta et al.^61^. After maintaining the culture of *S. litura* for three generations in the laboratory, the newly hatched larvae were used for conducting experiments.

### Bacterial isolation

The larvae and pupae of *S. litura* from third generation of laboratory culture were used for the isolation of culturable bacteria in the present study. Both larvae and pupae were sterilized with 70% (v/v) ethanol followed by washing with sterilized distilled water in order to remove the disinfectant. The larvae were dissected with sterilized micro scissors to remove the gut while the pupae were homogenised whole in 1.0 ml Phosphate Buffer Saline (PBS) solution (pH 7.0). The homogenised suspension was then serially diluted up to ten times and 100 µl of each diluted sample was then plated on Luria Bertani (LB) plates. The plates were incubated at 30 °C for 72 h for the observation of morphologically distinct colonies. The pure bacterial isolates were stored in 50% (w/v) glycerol at -80 °C. The identification of bacterial cultures was done by using various morphological, biochemical tests and molecular methods. On the basis of 16S rRNA gene sequencing the bacterial cultures were identified as *Staphylococcus gallinarum* (MW199124), *Bacillus safensis* (MW199274), *Enterococcus mundtii* (MW199120), *Enterococcus casseliflavus* (MW199276), *Staphylococcus sciuri* (MW199118), *Glutamicibacter halophytocola* (MW199121), *Corynebacterium terpenotabidum* (MW207679), *Serratia marcescens* (MW207987), and *Pantoea brenneri* (MW205745) (data submitted elsewhere).

### Preparation of bacterial suspension

Bacterial isolates were inoculated into LB broth and incubated at 30 °C for 48 h. After incubation the cultures was centrifuged at 4000 rpm at 4 °C for 10 min to obtain the pellet. The pellet was dissolved in sterile PBS solution and the bacterial density was measured at optical density (OD_600_) and adjusted to 1.89 (1.8 × 10^9^ cfu/ml approximately) and 10 ml of bacterial suspension was further used in bioassays as described by Eski et al.^29^ with some modifications.

### Screening bioassays

Second instar larvae of *S. litura* were used for screening the insecticidal activity of isolated bacterial cultures. The larvae were randomly selected and kept in rearing vials. The castor leaves were surface sterilized with 5% (v/v) NaOCl and washed with distilled water. The surface sterilized leaves of approximately 10cm^2^ were treated by dipping in 10 ml of bacterial suspension and were used in bioassays as described by Eski et al.^29^ with some modifications. After air drying at room temperature the treated leaves were kept in rearing vials containing larvae. Control group was fed on leaves dipped in PBS buffer only. The screening experiment for each bacterial culture was replicated 5 times with 10 larvae per replication (n = 50). During experiment the temperature and humidity conditions were maintained at 25 ± 2 °C and 65 ± 5% respectively. The diet was changed regularly after every 48 h till pupation and larval mortality was recorded.

### Dose response experiments

Based on higher larval mortality in *S. litura* due to *S. marcescens* and *E. mundtii*, both these cultures were used for dose response experiments. The concentration range for *S. marcescens* was, C_1_ = 2.6 × 10^8^ cfu/ml, C_2_ = 6.4 × 10^8^ cfu/ml, C_3_ = 1.6 × 10^9^ cfu/ml, C_4_ = 3.0 × 10^9^ cfu/ml and C_5_ = 5.2 × 10^9^ cfu/ml. The different concentrations used for cell suspension of *E. mundtii* were, C_1_ = 4.6 × 10^8^ cfu/ml, C_2_ = 8.9 × 10^8^ cfu/ml, C_3_ = 1.8 × 10^9^ cfu/ml, C_4_ = 3.4 × 10^9^ cfu/ml and C_5_ = 6.1 × 10^9^ cfu/ml (based on their OD_600_ values). The leaves dipped in PBS buffer only were fed to control group. The experiment was conducted in a similar manner as for screening bioassays. Observations were made daily on larval mortality, development period and adult emergence. The freshly emerged adults from all the treatments and control were transferred to oviposition jar in 2:1 ratio (2 females: 1 male) to observe the longevity and fecundity of adults. One oviposition jar represented one replicate and all the treatments were replicated thrice. Based on larval mortality data, lethal concentration (LC_50_) values for both the bacteria were determined by Probit analysis using the SPSS 20.0 statistical software.

### Nutritional analysis

In order to investigate the effect of bacteria on nutritional physiology of *S. litura*, the larvae were fed on castor leaves treated with different concentrations of *S. marcescens* and *E. mundtii* as mentioned above*.* The second instar larvae starved for 3–4 h were weighed individually and released in rearing vials containing treated and control leaves of known weight. The experiment was performed on 50 larvae for each concentration of both the bacterial cultures following the procedure of Datta et al.^61^. After 72 h of feeding, observations were made on larval weight, residual diet and faecal matter and overall change in each variable was compared with the last recorded value. Relative growth (RGR) and consumption rates (RCR) were calculated as *G*/*I* (*G* = change in larval dry weight/day and *I* = initial larval dry weight) and *C*/*I* (*C* = change in diet dry weight/day and *I* = initial larval dry weight) respectively. Both were calculated as mg mg^−1^ day^−1^. Index of food conversion efficiency (ECI) was calculated as 100 × *G*/*C*; where *G* = dry weight gain of insect and *C* = dry weight of food consumed. Approximate digestibility (AD) and efficiency of conversion of digested food (ECD) were calculated as *C* − *F*/*C* × 100 (where *C* = change in diet dry weight/day and *F* = dry weight of frass/day) and *G*/*C* − *F* × 100 (where *G* = change in larval dry weight/day, *C* = change in diet dry weight/day and *F* = dry weight of frass/day, respectively. All the nutritional indices were calculated as per Farrar et al.^62^_._

### Determination of effect of *S. marcescens* and *E. mundtii* on gut microflora of *S. litura*

To determine the effect of oral infection of bacteria on gut microbial composition of *S. litura*, pure cultures of *E. mundtii* and *S. marcescens* were inoculated in LB media. Second instar larvae were fed on leaves treated with LC_50_ values of *S. marcescens* and *E. mundtii.* After 96 h of feeding on treated leaves, ten infected larvae showing the symptoms of slow growth, reduction in size, black pigmentation on integument and control larvae were dissected separately to remove the gut. These larval guts of both infected and control larvae were then homogenized separately in 1 ml 0.1 M phosphate buffer (pH 7.0). A serial dilution of homogenized suspension was performed up to ten times and 100 µl of each dilution was spread on Luria Bertani (LB) agar plates. The plates were incubated for 48 h at 30 °C for appearance of bacterial colonies. The cfu/ml of different bacteria was calculated by plate count method. Each morphotype was purified by further streaking on LB plates. The bacterial isolates obtained were identified by using various morphological, biochemical tests and molecular methods.  Based on 16S rRNA gene sequencing these bacteria were identified as  * Enterococcus mundtii* (MW199120), *Enterococcus casseliflavus* (MW199276), *Serratia marcescens* (MW207987) and *Acinetobacter haemolyticus* (MW199127).

### Histological analysis

For histological studies the second instar larvae were fed on LC _50_ values of *S. marcescens* and *E. mundtii* cell suspension. In case of control, larvae were fed on leaves dipped in PBS buffer only. The experimental conditions were maintained at 25 ± 2 °C and 65 ± 5% respectively temperature and humidity respectively. After 96 h, both treated and control larvae were dissected aseptically and the gut was preserved in 10% formalin until processing of tissue. After fixation, the material was washed with distilled water in a tube and 30–90% grades of alcohol were used for progressive dehydration of tissue. After dehydration, the tissue from both control and treated larvae was fixed in paraffin wax. Thin ribbons from blocks were prepared using the microtome after solidification of wax blocks. These thin ribbons having gut sections were placed on slides coated with very thin layer of Mayer’s egg albumin and kept on warm hot plate at 40-45ºC temperature for equal spreading of wax. Again tissue section placed on slide was passed through 30–90% grades of alcohol in ascending and descending order. Then permanent staining of slides was done using the methodology of Verma and Srivastava^63^. Permanent mounting of tissue on slide was done using the DPX and covered with coverslip. After staining and mounting, the slides were observed under the microscope (Evos XL Core) at magnification  400X  for morphological changes in gut tissue.

### Growth of bacteria in larval hemolymph

The second instar larvae were fed on LC_50_ values of *S. marcescens* and *E. mundtii*. After 96 h of bacterial treatment, 100 µl of hemolymph was collected from both infected as well as control larvae. The hemolymph was serially diluted and spread on LB agar plates with the help of spreader. Plates were incubated at 30 °C and observed after 48 h upto 72 h for the appearance of bacterial colonies.

### Data analysis

To determine the differences among treated and control groups, the data on larval mortality, development period, adult emergence, adult deformities, reproductive potential and nutritional physiology were subjected to one way analysis of variance (ANOVA) followed by Tukey’s test at *p* ≥ 0.05. SPSS 20.0 software was used for statistical analysis.

### Ethics declarations

This article does not contain any studies involving humans/animals/plants that need approval from ethical committee.
